# Therapeutic Efficacy of pH-Dependent Release Formulation of Mesalazine on Active Ulcerative Colitis Resistant to Time-Dependent Release Formulation: Analysis of Fecal Calprotectin Concentration

**DOI:** 10.1155/2014/342751

**Published:** 2014-11-18

**Authors:** Kousaku Kawashima, Shunji Ishihara, Takafumi Yuki, Koji Onishi, Yoshinori Kushiyama, Hirofumi Fujishiro, Youichi Miyaoka, Mika Yuki, Yoshinori Komazawa, Takashi Tanimura, Hiroki Sonoyama, Yasumasa Tada, Ryusaku Kusunoki, Akihiko Oka, Nobuhiko Fukuba, Naoki Oshima, Ichiro Moriyama, Yoshikazu Kinoshita

**Affiliations:** ^1^Department of Internal Medicine II, Shimane University School of Medicine, 89-1 Enya-cho, Izumo 6938501, Japan; ^2^Division of Internal Medicine, Matsue Seikyo Hospital, 8-8-8 Nishitsuda, Matsue 6908522, Japan; ^3^Division of Gastroenterology, Matsue Red Cross Hospital, 200 Horo-cho, Matsue 6908506, Japan; ^4^Division of Gastroenterology, Shimane Prefectural Central Hospital, 4-1-1 Himebara-cho, Izumo 6938553, Japan; ^5^Division of Internal Medicine, Izumo City General Medical Center, 613 Nadabunn-cho, Izumo 6910003, Japan; ^6^Division of Gastroenterology, Matsue City Hospital, 32-1 Noshira-cho, Matsue 6908509, Japan; ^7^Division of Cancer Center, Shimane University Hospital, 89-1 Enya-cho, Izumo 6938501, Japan

## Abstract

*Purpose*. Few reports have compared the clinical efficacy of a pH-dependent release formulation of mesalazine (pH-5-ASA) with a time-dependent release formulation (time-5-ASA). We examined whether pH-5-ASA is effective for active ulcerative colitis (UC) in patients resistant to time-5-ASA. *Methods*. We retrospectively and prospectively analyzed the efficacy of pH-5-ASA in mildly to moderately active UC patients in whom time-5-ASA did not successfully induce or maintain remission. The clinical efficacy of pH-5-ASA was assessed by clinical activity index (CAI) before and after switching from time-5-ASA. In addition, the efficacy of pH-5-ASA on mucosal healing (MH) was evaluated in a prospective manner by measuring fecal calprotectin concentration. *Results*. Thirty patients were analyzed in a retrospective manner. CAI was significantly reduced at both 4 and 8 weeks after switching to pH-5-ASA. In the prospective study (*n* = 14), administration of pH-5-ASA also significantly reduced CAI scores at 4 and 8 weeks in these patients who were resistant to time-5-ASA. In addition, fecal calprotectin concentration was significantly decreased along with improvement in CAI after switching to pH-5-ASA. *Conclusions*. Our results suggest that pH-5-ASA has clinical efficacy for mildly to moderately active patients with UC in whom time-5-ASA did not successfully induce or maintain remission.

## 1. Introduction

Mesalazine, a 5-aminosalicylic acid (5-ASA) compound, is widely recognized as the first line drug for induction therapy for mildly to moderately active ulcerative colitis (UC), because of its efficacy and safety [1–6]. Several formulations of oral mesalazine are available and primarily differentiated by the means of delivering active mesalazine to the colon. The time-dependent release formulation of mesalazine (time-5-ASA) is coated with ethyl cellulose and begins to release 5-ASA in the duodenum; then the release is continued throughout the large intestine [7, 8]. On the other hand, the pH-dependent release formulation of mesalazine (pH-5-ASA) is coated with Eudragit-S and starts to release the drug in the terminal ileum or cecum, since the coating film breaks down at pH 7 or higher [9, 10]. High-dose treatments with these formulations are effective for induction of remission in patients with UC as compared to conventional dosages.

The therapeutic efficacy of oral mesalazine preparations for UC patients has been reported to be dependent on the efficiency of delivering active mesalazine to the colon. A recent study revealed that the mucosal mesalazine concentration in the sigmoid colon in patients treated with pH-5-ASA was higher than that in patients treated with time-5-ASA [11]. Those findings suggest that pH-5-ASA may be effective for active UC in patients for whom time-5-ASA did not successfully induce or maintain remission. However, it remains unknown whether switching from time-5-ASA to pH-5-ASA contributes to therapeutic efficacy in those patients.

Mucosal healing (MH) is currently regarded as an important treatment goal in patients with inflammatory bowel diseases (IBD) [12]. Although an endoscopy examination is recognized as the most reliable method for evaluating MH that is relatively invasive and sometimes painfull. Fecal calprotectin has been proposed as a noninvasive reliable surrogate marker for MH, because of its strong correlation with endoscopically proven UC activity [13–15], and the clinical efficacy of fecal calprotectin for evaluating MH has been shown to be superior as compared to other noninvasive laboratory tests including erythrocyte sedimentation (ESR) and serum C-reactive protein (CRP) levels [14, 16].

The aim of this study was to investigate whether pH-5-ASA is effective for mild to moderate UC in patients for whom time-5-ASA did not successfully induce or maintain remission. We performed retrospective and prospective studies to examine the effect of switching to pH-5-ASA from time-5-ASA to decrease clinical disease activity and induce MH.

## 2. Methods

### 2.1. Retrospective Study

#### 2.1.1. Patients

This retrospective study was conducted from January 2010 to August 2011 at 6 hospitals and 3 clinics in Japan. Eligible subjects were UC patients aged 18 years or older with a Rachmilewitz clinical activity index (CAI) [17] greater than 5 and/or rectal bleeding. In addition, the mesalazine formulation used in those patients was switched from time-5-ASA (Pentasa) to pH-5-ASA (Asacol) because of exacerbation or insufficient efficacy under administration of time-5-ASA (Pentasa) at greater than 2.25 g/day for at least 2 months. Patients were excluded if they had received oral salazosulfapyridine, corticosteroids, immunomodulatory drugs, or biologics or had undergone leukocytapheresis. Patient demographics, age, sex, disease extent, and doses of time-5-ASA and pH-5-ASA were also investigated.

#### 2.1.2. Study Design and Statistical Analysis

Change in CAI was retrospectively assessed at 0, 4, and 8 weeks after switching to pH-5-ASA. The frequency of usage of a mesalazine-based enema during the prior 7 days in weeks 0 and 8 was also investigated. Clinical assessment was evaluated after 8 weeks as follows: remission: CAI 0 or 1, improvement: CAI decreased by more than 2 points, no change: CAI not changed or decreased by 1 point only, and exacerbation: CAI increased or increased in frequency of mesalazine-based enema use. Changes in CAI were statistically analyzed using Wilcoxon's signed rank test. *P* < 0.05 was considered to be statistically significant.

### 2.2. Prospective Study

This open-label prospective study was conducted at Shimane University Hospital and Matsue Seikyo Hospital from August 2011 to July 2013. The ethics committee at each institution approved the protocol and all patients gave written informed consent in accordance with the Helsinki Declaration.

#### 2.2.1. Patient Selection

UC patients aged 18 years and older with mild to moderate activity (CAI between 5 and 11) that was exacerbated during maintenance therapy using time-5-ASA (Pentasa) greater than 2.25 g/day were enrolled as subjects. Exclusion criteria were as follows: treatment with oral salazosulfapyridine, corticosteroids, immunomodulatory drugs, or biologics for at least 3 months; receiving leukocytapheresis therapy; severe active UC (CAI 12 or more); positive results of stool culture for bacterial pathogens; current renal or hepatic disease; or medical contraindication for study participation. Patient demographics, age, sex, disease extent, duration and dose of time-5-ASA, and severity were investigated.

#### 2.2.2. Study Schedule

In patients who met the inclusion criteria, pH-5-ASA was administrated instead of time-5-ASA for 8 weeks. A daily dose of 2.25 g of time-5-ASA was switched to 2.4 g of pH-5-ASA, while a daily dose above 2.25 g of time-5-ASA was switched to 3.6 g of pH-5-ASA. Patients using a mesalazine-based enema were allowed to continue that treatment at the same dosage and frequency during the study. Nonsteroidal anti-inflammatory drugs and antidiarrheal and antispasmodic medications were not allowed during the study.

In order to determine CAI, the frequency of bowel movements, bloody stools, and abdominal pain were monitored at weeks 0, 4, and 8. Peripheral blood samples were collected for measurement of complete blood count, ESR, and high sensitive CRP (hsCRP). Patients who required additional treatments based on physician assessment were withdrawn from the study at that time.

#### 2.2.3. Measurement of Fecal Calprotectin

Fecal samples were collected twice at weeks 0 and 8. In patients withdrawn from the study due to exacerbation, fecal samples were collected on the day of study discontinuation. Fecal samples were stored in a freezer at −20°C until measurements. The calprotectin concentration was determined using a quantitative enzyme-linked immunosorbent assay (PhiCal, Immundiagnostik, Germany).

#### 2.2.4. Assessment and Statistical Analysis

The primary endpoint for the study was clinical efficacy after switching to pH-5-ASA treatment. Changes in CAI scores (at weeks 4 and 8) were statistically analyzed using Wilcoxon's signed rank test. Clinical assessment was evaluated at week 8 as follows: remission: CAI 0 or 1, improvement: CAI decreased by more than 2 points, no change: CAI not changed and decreased by 1 point only, and exacerbation: CAI increased. The secondary endpoint was a decrease in fecal calprotectin concentration. Changes in fecal calprotectin concentration were analyzed using Wilcoxon's singed rank test. *P* < 0.05 was considered to be statistically significant.

## 3. Results

### 3.1. Retrospective Study

**Figure 1:**
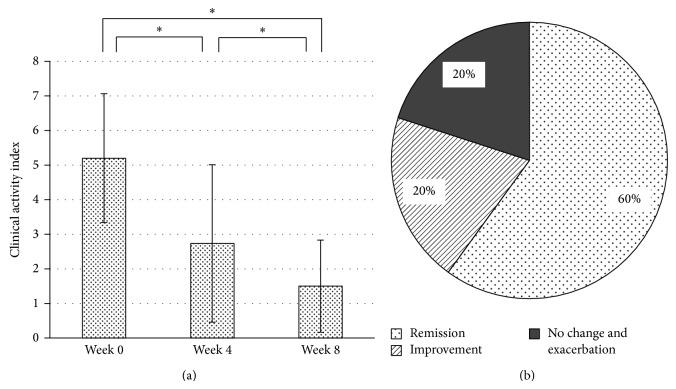
Results of retrospective study. (a) Changes in clinical activity index. (b) Clinical assessments at 8 weeks. ^*^ *P* < 0.001.

Thirty patients who met the inclusion criteria were enrolled and their demographics are shown in Table 1. The mean dose of time-5-ASA was 3025 ± 839.1 mg/day, while that of pH-5-ASA after switching was 3120 ± 597.9 mg/day. Changes in mean CAI are presented in Figure 1(a). Mean CAI at week 0 was 5.20 ± 1.84, while that at weeks 4 and 8 was 2.73 ± 2.27 and 1.50 ± 1.33, respectively. CAI was significantly reduced at both weeks 4 and 8 (*P* < 0.001) after switching to pH-5-ASA. Mean CAI in 12 patients who switched from time-5-ASA at 4 g/day was also significantly reduced (before, 5.08 ± 1.31; 4 weeks, 2.50 ± 2.02; 8 weeks, 1.58 ± 1.08). Clinical assessment findings at week 8 are shown in Figure 1(b). Twenty-four patients (80.0%) showed improvement or remission.

### 3.2. Prospective Study

#### 3.2.1. Patient Characteristics

Fourteen patients who met the inclusion criteria were enrolled and their baseline characteristics are shown in Table 2. The mean age at entry was 45.1 ± 16.6 years old. The daily dose of time-5-ASA before switching to pH-5-ASA was 2.25 g in 10 and 3.0 g in 4 patients. Clinical severity at entry was mildly active in 10 patients and moderately active in 4 patients. No patient had received a mesalazine-based enema prior to entry.

#### 3.2.2. Clinical Efficacy

**Figure 2:**
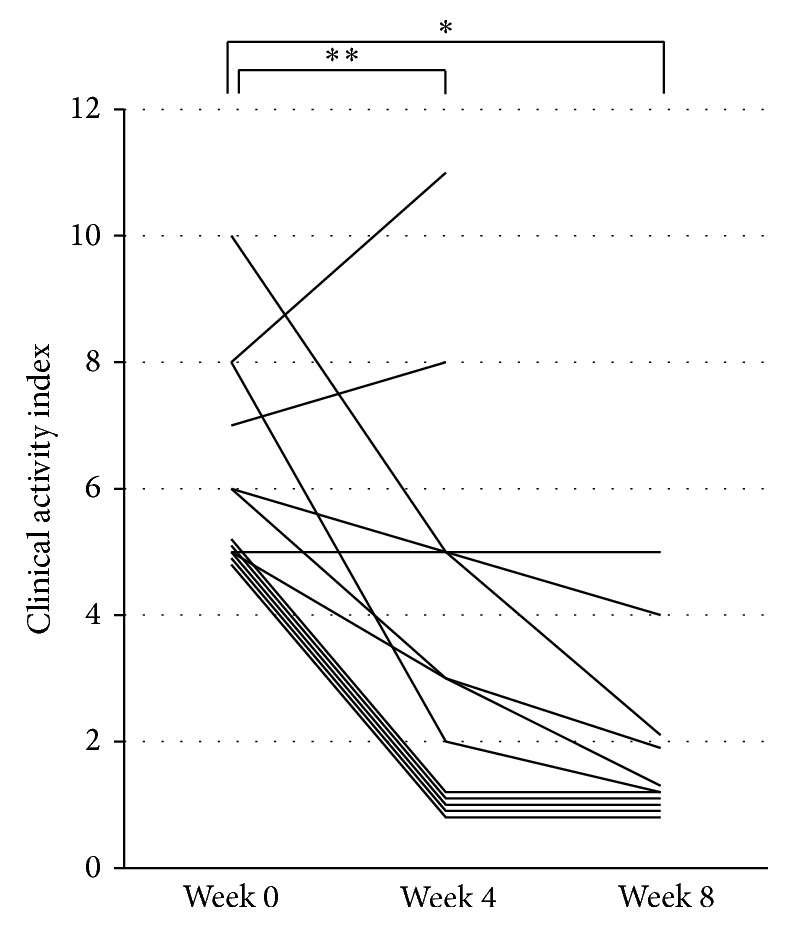
Changes in clinical activity index in prospective study. ^**^ *P* = 0.009 and ^*^ *P* = 0.002.

Of the 14 patients enrolled, 1 male (case 14) was excluded from analysis of efficacy because of insufficient compliance to the protocol. Therefore, 13 patients were analyzed for clinical efficacy. Of those, 11 continued the pH-5-ASA administration for 8 weeks, while 2 patients (cases 9 and 10) were withdrawn from the study because their physicians decided that additional treatments were needed due to exacerbation at week 4. Changes in CAI for each patient are presented in Figure 2. Mean CAI was 6.15 ± 1.63 at week 0, 3.62 ± 3.12 at week 4, and 1.82 ± 1.40 at week 8. CAI scores at weeks 4 and 8 were significantly reduced as compared to that at entry (week 4, *P* = 0.009; week 8, *P* = 0.002). Clinical assessments at week 8 showed remission in 7, improvement in 3, no change in 1, and exacerbation in 2; thus 10 patients (76.9%) showed improvement or remission. No adverse effects were observed during this study.

#### 3.2.3. Changes in Fecal Calprotectin Concentration and Serum hsCRP Level

**Figure 3:**
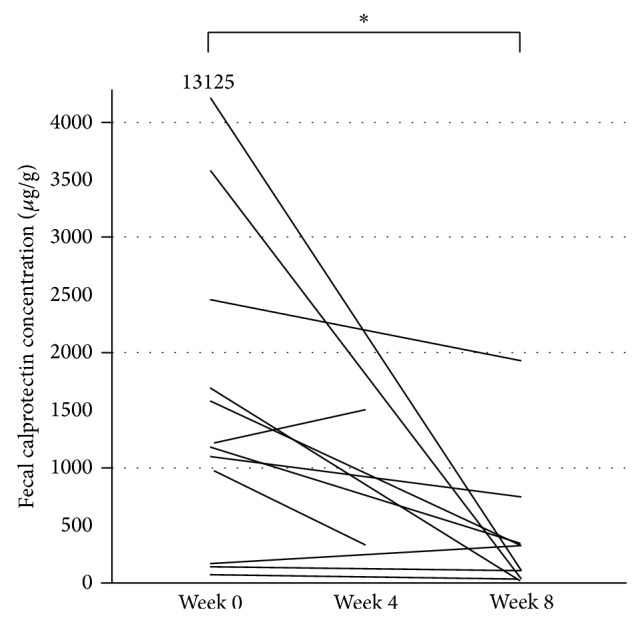
Changes in fecal calprotectin concentration in prospective study. ^*^ *P* = 0.012.

Of the 13 patients analyzed, 1 (case 13) did not send in the fecal sample and 2 withdrew. Thus, the change in fecal calprotectin concentration at week 8 was analyzed in 10 patients. Changes in fecal calprotectin concentration for each patient are presented in Figure 3. Mean fecal calprotectin concentration at entry (*n* = 12) was 2288.6 ± 3562.9 *μ*g/g (median 1194.6), while that at week 8 (*n* = 10) was 395.6 ± 581.3 *μ*g/g (median 217.5). The fecal calprotectin concentration at week 8 was significantly reduced as compared to that at entry (*P* = 0.012). Fecal samples from the 2 withdrawn patients (cases 9 and 10) were collected at week 4 and the fecal calprotectin concentration in that from case 9 was not reduced, while that from case 10 was slightly reduced. Mean hsCRP at entry was 0.459 ± 0.576 g/L, while that at week 8 was 0.184 ± 0.300 g/L. The decrease in hsCRP was not statistically significant (*P* = 0.084).

## 4. Discussion

Results of both the retrospective and prospective studies clarified the therapeutic efficacy of switching to pH-5-ASA for mildly to moderately active UC patients in whom time-5-ASA did not successfully induce or maintain remission. The therapeutic effect of pH-5-ASA for MH was clearly shown by a decrease in fecal calprotectin concentration in the prospective study. Our results suggest that switching to pH-5-ASA is a viable therapeutic option for UC patients resistant to time-5-ASA.

Although dose-related efficacies of various oral mesalazine formulations for induction of remission in active UC patients have been reported [18–22], there are few direct comparisons of those based on the different systems for delivery of active mesalazine to the colon. A double-blind randomized trial that compared the efficacy of 2 different mesalazine formulations was performed in Japan by Ito et al. [23], which showed that pH-5-ASA at 2.4 g/day was equally effective as time-5-ASA at 2.25 g/day for patients with active UC. However, to the best of our knowledge, there is no report regarding the clinical efficacy of pH-5-ASA in active UC patients resistant to time-5-ASA.

Oral mesalazine exerts its anti-inflammatory effect directly on inflamed mucosa in the large intestine, while therapeutic efficacy is thought to depend on colonic mucosal concentrations of the drug [24, 25]. Recently, D'Incà et al. showed that the mean mucosal concentration of mesalazine was significantly higher in pH-5-ASA-treated patients as compared to time-5-ASA-treated patients [11]. In this regard, we speculated that pH-5-ASA may be effective for UC patients who show resistance to time-5-ASA.

Initially, we retrospectively analyzed the efficacy of pH-5-ASA in UC patients with mild to moderate activity in whom time-5-ASA did not successfully induce or maintain remission. Our findings revealed that an 8-week administration of pH-5-ASA significantly reduced CAI and improved clinical assessment regardless of the extent of UC. In addition, efficacy was also clearly shown in patients who switched from high-dose time-5-ASA (4 g/day). To confirm the results of our retrospective study, we then performed a prospective study to investigate the efficacy of pH-5-ASA in active UC patients resistant to time-5-ASA. The clinical efficacy of switching to pH-5-ASA was also shown in those patients. Taken together, the present results suggest that the efficacy of mesalazine in a certain population of UC patients is dependent on the type of formulation, which might be associated with efficient delivery of active mesalazine to the colon. In this regard, we speculated that switching to pH-5-ASA may be more effective in left-sided UC or proctitis cases. However, the efficacy noted with switching to pH-5-ASA was not related to disease extent in our retrospective and prospective studies. In addition, we did not find an association between disease severity and efficacy after switching to pH-5-ASA, though that result may be related to the small sample size.

Recently, MH has been proposed as a major therapeutic goal in clinical trials of UC patients [12] and has shown to be associated with higher clinical response and lower risk of clinical relapse [26–28]. A recent meta-analysis conducted by Römkens et al. demonstrated that an appropriate dose of oral mesalazine formulation successfully leads to MH along with induction of clinical remission in active UC patients [29]. In the present prospective study, we measured fecal calprotectin concentrations for evaluating the efficacy of pH-5-ASA on MH. Those results showed that pH-5-ASA decreased fecal calprotectin concentration in UC patients who demonstrated exacerbation during maintenance therapy with time-5-ASA and suggested that switching to pH-5-ASA might have contributed to MH by efficiently delivering active mesalazine to inflamed mucosa. In contrast, serum hsCRP level was not significantly decreased in those patients after receiving pH-5-ASA treatment. Previous studies have demonstrated that endoscopically evident activity in UC is strongly correlated with fecal calprotectin concentration as compared to CRP [14, 30]. Thus, CRP does not seem to be adequately sensitive for detecting low grade inflammation in assessment of MH.

Our findings suggest that switching to pH-5-ASA is a viable option as induction therapy for active UC patients who show resistance to time-5-ASA. However, it must be kept in mind that a delay of appropriate treatment may increase the severity of UC; thus attention must be given to determine whether an alternative treatment (e.g., corticosteroids) or switching to pH-5-ASA should be used.

Our prospective study has several limitations. First, it was open label and there was no comparison made using a control group. A placebo effect may have existed in assessment of clinical efficacy. Second, the sample size was small. Third, selection bias may have occurred when we chose to enroll mildly to moderately active UC patients. In particular, disease severity in UC with moderate activity is distributed over a wide range. Finally, the concentration of mesalazine in colonic mucosa before and after switching the mesalazine formulation was not measured, though we speculate that the efficacy of pH-5-ASA may be dependent on efficient delivery of active mesalazine to the colon. To further confirm the efficacy of switching to another oral mesalazine formulation, a study considering these limitations should be conducted.

In conclusion, this is the first report to clarify the efficacy of pH-5-ASA for UC patients with mild to moderate activity who show resistance to time-5-ASA. Furthermore, we revealed that pH-5-ASA contributed to MH by confirming a decrease in fecal calprotectin concentration in those patients. Switching to pH-5-ASA may be a viable therapeutic option for induction therapy in UC patients resistant to pH-5-ASA.

## Figures and Tables

**Table 1:** Demographics of 30 eligible patients in retrospective study.

Age at entry (years)	46.1 ± 14.8
Gender (M/F)	12/18
Disease extent	
Extensive	5
Left-sided	12
Proctitis	13
Dose of prior mesalazine (Pentasa)	
2250 mg	15
3000 mg	3
4000 mg	12
Dose of switched mesalazine (Asacol)	
2400 mg	12
3600 mg	18

**Table 2:** Baseline characteristics of 14 eligible patients in prospective study.

Patient number	Age (years)	Gender	Disease extent	Dose of prior mesalazine (mg)	Severity	Rachmilewitz CAI
1	27	M	Extensive	3000	Mild	5
2	73	F	Left-sided	3000	Mild	6
3	37	M	Left-sided	2250	Mild	5
4	39	F	Extensive	3000	Mild	5
5	63	F	Extensive	2250	Moderate	10
6	33	F	Left-sided	2250	Mild	5
7	59	F	Extensive	3000	Mild	6
8	40	M	Extensive	2250	Mild	5
9	27	M	Extensive	2250	Moderate	8
10	57	M	Left-sided	2250	Moderate	7
11	27	F	Proctitis	2250	Mild	5
12	72	M	Extensive	2250	Moderate	8
13	31	F	Proctitis	2250	Mild	5
14	47	M	Left-sided	2250	Mild	5
